# From class effects to specificity FAERS evidence and network mapping of adverse events in NSCLC targeted therapy

**DOI:** 10.1097/JS9.0000000000004704

**Published:** 2026-01-21

**Authors:** Jinsheng Yu, Minqi Zhu, Yiwen Zhu, Qijin Shu

**Affiliations:** a Department of Oncology, The First Affiliated Hospital of Zhejiang Chinese Medical University (Zhejiang Provincial Hospital of Traditional Chinese Medicine), Hangzhou, Zhejiang, China; b The First School of Clinical Medicine, Zhejiang Chinese Medical University, Hangzhou, Zhejiang, China

**Keywords:** FDA Adverse Event Reporting System, non-small cell lung cancer, pharmacovigilance, targeted anticancer drugs

## Abstract

**Background:**

Whether targeted therapies for non-small cell lung cancer (NSCLC) share mechanism-driven class toxicities or mainly exhibit drug-specific risks remains unclear in real-world practice.

**Methods:**

We analyzed reports from the U.S. FDA Adverse Event Reporting System (FAERS, 2004–2025) for 14 Food and Drug Administration (FDA)-approved agents across five classes: EGFR, ALK, ROS1, RET tyrosine kinase inhibitors (TKIs), and a KRAS G12C inhibitor. Adverse events (AEs) were standardized to MedDRA v27.0 preferred terms (PTs). Primary PT-level safety signals were defined based on concordance across four disproportionality methods: proportional reporting ratio (PRR; PRR ≥ 2, x^2^ ≥ 4, and ≥3 reports), reporting odds ratio (ROR; lower 95% confidence interval bound ROR_025_ > 1), Bayesian confidence propagation neural network (BCPNN; IC_025_ > 0), and the multi-item gamma Poisson shrinker (MGPS; EB05 ≥ 2). Cross-drug structure was evaluated via a Jaccard-based similarity network.

**Results:**

Among 34 948 individuals, per-drug signal counts ranged from 3 to 113. EGFR-TKIs were enriched for mucocutaneous and gastrointestinal events; ALK-TKIs for metabolic and laboratory abnormalities and selected cardiac findings; and RET-TKIs for hepatotoxicity and hypertension. Osimertinib showed prominent electrocardiographic signals (e.g., QT prolongation); lorlatinib exhibited a distinctive dyslipidemia signature. Brigatinib and crizotinib aligned with creatine kinase elevation and visual effects, respectively. No single PT occurred across all 14 drugs. Recurrent cross-class PTs included increased blood pressure, QT prolongation, dry skin, and edema. The similarity network revealed tight within-class modules (EGFR, ALK), a binary RET pair, and peripheral placement of repotrectinib and adagrasib, indicating limited overlap of their AE profiles.

**Conclusion:**

This first NSCLC-focused FAERS comparison integrating four-method signal detection with network analysis delineates reproducible class effects superimposed by drug-specific toxicities. Findings support tailored monitoring (e.g., dermatologic care for EGFR-TKIs; ECG/electrolytes for osimertinib; lipid/CK surveillance for ALK-TKIs; blood pressure/liver testing for RET-TKIs) to inform risk-aware first-line decisions.

## Introduction

Non-small cell lung cancer (NSCLC) constitutes the majority of lung cancers and remains a leading cause of cancer-related mortality worldwide^[1,2]^, with a highly heterogeneous molecular landscape and prognosis. Abnormal expression of actionable molecular biomarkers is closely linked to adverse survival outcomes[3], making the trade-off between efficacy and tolerability in first-line therapy particularly critical. The rise of molecular subtyping and targeted therapy has disrupted “one-size-fits-all” care: inhibitors of EGFR, ALK, ROS1, RET, and KRAS (G12C) have entered the frontline setting and substantially improved survival in selected populations^[4–13]^. Yet a pressing reality persists – alongside improved efficacy, treatment-related adverse events (AEs) and overall safety burden continue to accumulate, driving dose reductions, switching, and discontinuation, and complicating real-time clinical decision-making.

**HIGHLIGHTS**

- First non-small cell lung cancer (NSCLC)-focused pharmacovigilance map for 14 targeted agents in U.S. FDA Adverse Event Reporting System (2004–2025).
- Signals were defined by concordance across PRR, ROR, BCPNN and MGPS, and integrated with Jaccard-based similarity network (heatmap, clustering, MDS).
- Distinguishes mechanism-driven class effects from drug-specific toxicities.
- Identifies reproducible cross-class AEs (increased blood pressure, QT prolongation, dry skin, and edema).

Evidence for drug-related AEs is uneven both within and across targeted classes. Cutaneous and gastrointestinal AEs of EGFR tyrosine kinase inhibitors (TKIs) are well recognized and often manageable^[14,15]^, but serious toxicities such as thrombosis with osimertinib[16], pneumatosis intestinalis^[17,18]^, and altered mental status with erlotinib[19] have also been reported. For ALK inhibitors, beyond common hepatic and metabolic abnormalities[20], case reports suggest venous thrombosis with crizotinib[21] and seizures with ceritinib[22], which are not consistently emphasized in product labeling. Safety profiles of RET and KRAS (G12C) inhibitors remain less well defined because of their shorter time in clinical use^[23,24]^. Traditional clinical trials have limited follow-up and focus mainly on efficacy endpoints, making them less suitable for detecting rare or delayed systemic AEs and for distinguishing mechanism-based class effects from drug-specific risks.

The U.S. FDA Adverse Event Reporting System (FAERS)-based pharmacovigilance studies have, to some extent, helped to address these gaps. Oshima *et al* reported that when EGFR-TKIs are used in combination with or sequentially after nivolumab, the reporting proportion of EGFR-TKI-associated interstitial pneumonitis increased significantly[25]. Post-marketing analyses of ALK inhibitors have suggested organ-specific AE profiles and have identified pneumothorax, pulmonary arterial hypertension and myasthenia gravis as potential signals of regulatory concern, but they treated “lung cancer” as a single entity without distinguishing NSCLC[20]. FAERS-based studies of KRAS G12C inhibitors have also reported multiple treatment-related AEs but did not restrict analyses to specific indications[26]. Overall, these studies provide important post-marketing safety information for targeted therapies, but most are limited to a single therapeutic spectrum or pan-cancer cohorts and still lack a systematic comparison, within a unified NSCLC indication, of “class effects” versus “drug-specific” toxicities across multiple molecular targets.

To address these gaps, we focused on NSCLC and used the FAERS to delineate post-marketing safety across five targeted classes. AEs were standardized to MedDRA, compared at the System Organ Class (SOC) and preferred term (PT) levels, and detected using multiple disproportionality metrics for robust signal identification. We then built a drug–positive-PT similarity network to test an intuitive hypothesis: agents sharing a molecular target cluster by AE patterns, whereas cross-target overlap is limited and weaker. By integrating signal detection with network-level comparisons across 14 Food and Drug Administration (FDA)-approved agents, this study seeks to distinguish class effects from drug-specific AEs, complement trial and case-report findings, and provide actionable real-world evidence for risk monitoring, individualized treatment decisions, and optimization of first-line strategies. In accordance with the TITAN Guidelines 2025 (Supplemental Digital Content Material, available at: http://links.lww.com/JS9/G620) governing the declaration and use of artificial intelligence, we confirm that no AI tools were utilized in the data generation, analysis, or manuscript preparation of this study[27].

## Materials and methods

### Data source

We performed a retrospective pharmacovigilance disproportionality analysis using FAERS. Source data were downloaded from the FDA website and included seven datasets: demographics/administrative information (DEMO), drug information (DRUG), AEs (REAC), patient outcomes (OUTC), report sources (RPSR), therapy start/stop dates (THER), and indications (INDI). FAERS is publicly accessible; all records are anonymized and de-identified. Institutional review board approval and informed consent were not required.

### Procedures

We retrieved FAERS reports for 14 FDA-approved targeted anticancer agents with indications including NSCLC between 1 January 2004 and 30 June 2025. The indication terms used to define NSCLC in FAERS are listed in Supplemental Digital Content Table 2a, available at: http://links.lww.com/JS9/G623 and jointly delineate the NSCLC population. Drug classes and individual agents were: EGFR-TKIs (gefitinib, erlotinib, afatinib, dacomitinib, osimertinib), ALK-TKIs (crizotinib, ceritinib, alectinib, brigatinib, lorlatinib), ROS1-TKI (repotrectinib), RET-TKIs (pralsetinib, selpercatinib), and the KRAS (G12C) inhibitor adagrasib. Drug identification used both generic and brand names. AEs were standardized to MedDRA v27.0 PTs and mapped to SOCs[28]. De-duplication followed FDA guidance: for records with the same CASEID, only the report with the most recent PRIMARYID was retained; for records with identical CASEID and FDA_DT, the record with the larger PRIMARYID was retained, followed by a consistency check. FAERS encodes drug roles as primary suspect, secondary suspect, concomitant, or interacting. To maximize specificity, the primary analysis included only reports in which the study drug was designated as the primary suspect. Extracted covariates included sex, age, weight, indication, seriousness, outcome, reporter type, geographic region, and report date.

### Statistical analysis

#### Disproportionality analysis

Disproportionality analysis is a standard pharmacovigilance approach to identify drug–event signals. We jointly applied two frequentist methods – the proportional reporting ratio (PRR)[29] and the reporting odds ratio (ROR)[30] – and two Bayesian methods – the Bayesian confidence propagation neural network (BCPNN)[31] and the multi-item gamma–Poisson shrinker (MGPS)[32]. PRR and ROR are derived from 2 × 2 contingency tables and provide intuitive measures of disproportionality. BCPNN and MGPS are empirical Bayes methods suitable for sparse data and yield more stable estimates. For BCPNN, we used the information component (IC) and its 95% confidence interval lower bound (IC_025_); IC_025_ > 0 indicated a positive signal. For MGPS, we used the empirical Bayes geometric mean (EBGM) and its 90% credibility interval lower bound EB05; EB05 ≥ 2 was taken as a signal, in line with FDA guidance[33].

To balance sensitivity and specificity and to remain consistent with prior pharmacovigilance practice^[24,34,35]^, a drug–event pair was classified as a key PT-level signal only when all four metrics met predefined thresholds. Exact formulas and criteria are provided in the Supplemental Digital Content Methods, available at: http://links.lww.com/JS9/G620and Supplemental Digital Content Table 1, available at: http://links.Lww.Com/JS9/G622

**Table 1:** Baseline characteristics of NSCLC FAERS reports after multiple imputation, by targeted agent.

Indicator	Gefitinib	Erlotinib	Afatinib	Dacomitinib	Osimertinib	Crizotinib	Ceritinib	Alectinib	Brigatinib	Lorlatinib	Repotrectinib	Pralsetinib	Selpercatinib	Adagrasib
AE	N = 3982	N = 8820	N = 3372	N = 190	N = 7967	N = 2781	N = 1324	N = 2978	N = 1362	N = 1344	N = 41	N = 344	N = 310	N = 133
Sex														
Female	2415 (60.6%)	4619 (52.4%)	2074 (61.5%)	89 (46.8%)	5168 (64.9%)	1591 (57.2%)	702 (53%)	1738 (58.4%)	787 (57.8%)	747 (55.6%)	27 (65.9%)	197 (57.3%)	179 (57.7%)	54 (40.6%)
Male	1567 (39.4%)	4201 (47.6%)	1298 (38.5%)	101 (53.2%)	2799 (35.1%)	1190 (42.8%)	622 (47%)	1240 (41.6%)	575 (42.2%)	597 (44.4%)	14 (34.1%)	147 (42.7%)	131 (42.3%)	79 (59.4%)
Weight														
Mean (SD)	61.5 (16.5)	62.1 (17.7)	64.8 (20.8)	61.4 (12.2)	58.8 (16.1)	65.5 (17.6)	61.2 (16.9)	67.3 (17.6)	65 (18.4)	69.3 (22.6)	62 (21)	73.1 (19.6)	62.7 (12.1)	70.3 (18.3)
Median (Q1–Q3)	59.9 (50–70)	59 (50–70.3)	62 (51–74)	62 (52.4–67)	57 (47–67)	63 (53–75)	60 (49.2–70)	65 (56–75)	61.5 (52.5–71.6)	65 (55–79.9)	58.1 (45–64.4)	68 (60.7–84)	61 (54–71.6)	67 (56–-87)
<50 kg	316 (7.9%)	313 (3.5%)	266 (7.9%)	17 (8.9%)	607 (7.6%)	199 (7.2%)	68 (5.1%)	93 (3.1%)	37 (2.7%)	99 (7.4%)	2 (4.9%)	6 (1.7%)	14 (4.5%)	6 (4.5%)
>100 kg	21 (0.5%)	60 (0.7%)	26 (0.8%)	NA	28 (0.4%)	44 (1.6%)	13 (1.0%)	41 (1.4%)	13 (1.0%)	31 (2.3%)	1 (2.4%)	7 (2.0%)	NA	2 (1.5%)
50 ~ 100 kg	892 (22.4%)	1314 (14.9%)	726 (21.5%)	73 (38.4%)	1153 (14.5%)	855 (30.7%)	300 (22.7%)	806 (27.1%)	261 (19.2%)	380 (28.3%)	6 (14.6%)	57 (16.6%)	66 (21.3%)	45 (33.8%)
Age														
Mean (SD)	66 (11.9)	64.1 (16.5)	62.8 (17.2)	61.8 (15.8)	67.6 (13.6)	59.6 (16.6)	57 (13)	53.3 (22.4)	43.9 (29)	53.7 (20.3)	32.9 (34.1)	60.1 (12.3)	63.6 (12.8)	65.8 (10.6)
Median (Q1–Q3)	67 (58–75)	67 (58–74.2)	66 (57–73)	64 (56.2–72)	69 (60–77)	62 (50–71)	57 (48–66)	57.5 (44–69)	54 (0.8–67)	57 (45–68)	33 (0.7–64)	60 (52.8–69.2)	65 (55–75)	66 (60–73)
< 18	NA	84 (1.0%)	43 (1.3%)	3 (1.6%)	24 (0.3%)	34 (1.2%)	2 (0.2%)	176 (5.9%)	162 (11.9%)	27 (2.0%)	9 (22.0%)	NA	NA	NA
>85	112 (2.8%)	189 (2.1%)	70 (2.1%)	2 (1.1%)	331 (4.2%)	47 (1.7%)	8 (0.6%)	25 (0.8%)	12 (0.9%)	10 (0.7%)	NA	NA	7 (2.3%)	NA
18 ~ 64.9	1391 (34.9%)	2512 (28.5%)	1051 (31.2%)	84 (44.2%)	1825 (22.9%)	1295 (46.6%)	714 (53.9%)	1241 (41.7%)	456 (33.5%)	666 (49.6%)	10 (24.4%)	100 (29.1%)	96 (31.0%)	33 (24.8%)
65 ~ 85	1719 (43.2%)	3467 (39.3%)	1392 (41.3%)	87 (45.8%)	3085 (38.7%)	992 (35.7%)	302 (22.8%)	791 (26.6%)	322 (23.6%)	369 (27.5%)	9 (22.0%)	70 (20.3%)	111 (35.8%)	32 (24.1%)
Reporter type														
Consumer	657 (16.5%)	3917 (44.4%)	545 (16.2%)	86 (45.3%)	1676 (21.0%)	721 (25.9%)	434 (32.8%)	611 (20.5%)	419 (30.8%)	362 (26.9%)	7 (17.1%)	141 (41.0%)	195 (62.9%)	12 (9.0%)
Health professional	249 (6.3%)	489 (5.5%)	218 (6.5%)	27 (14.2%)	731 (9.2%)	109 (3.9%)	81 (6.1%)	280 (9.4%)	77 (5.7%)	155 (11.5%)	2 (4.9%)	58 (16.9%)	23 (7.4%)	26 (19.5%)
Pharmacist	119 (3.0%)	263 (3.0%)	96 (2.8%)	7 (3.7%)	566 (7.1%)	144 (5.2%)	36 (2.7%)	205 (6.9%)	85 (6.2%)	87 (6.5%)	7 (17.1%)	17 (4.9%)	13 (4.2%)	32 (24.1%)
Physician	1559 (39.2%)	2608 (29.6%)	2249 (66.7%)	70 (36.8%)	3625 (45.5%)	1538 (55.3%)	526 (39.7%)	1858 (62.4%)	716 (52.6%)	713 (53.1%)	25 (61.0%)	128 (37.2%)	79 (25.5%)	63 (47.4%)
other	1398 (35.1%)	1543 (17.5%)	264 (7.8%)	NA	1369 (17.2%)	269 (9.7%)	247 (18.7%)	24 (0.8%)	65 (4.8%)	27 (2.0%)	NA	NA	NA	NA
Outcome														
Death	402 (10.1%)	2288 (25.9%)	765 (22.7%)	90 (47.4%)	2599 (32.6%)	869 (31.2%)	459 (34.7%)	250 (8.4%)	306 (22.5%)	364 (27.1%)	4 (9.8%)	56 (16.3%)	47 (15.2%)	31 (23.3%)
Disability	17 (0.4%)	30 (0.3%)	28 (0.8%)	2 (1.1%)	52 (0.7%)	18 (0.6%)	8 (0.6%)	18 (0.6%)	2 (0.1%)	12 (0.9%)	3 (7.3%)	2 (0.6%)	1 (0.3%)	6 (4.5%)
Hospitalization	266 (6.7%)	1095 (12.4%)	910 (27.0%)	31 (16.3%)	1583 (19.9%)	644 (23.2%)	336 (25.4%)	468 (15.7%)	314 (23.1%)	312 (23.2%)	8 (19.5%)	127 (36.9%)	87 (28.1%)	50 (37.6%)
Life-threatening	41 (1.0%)	88 (1.0%)	115 (3.4%)	6 (3.2%)	274 (3.4%)	101 (3.6%)	21 (1.6%)	45 (1.5%)	19 (1.4%)	31 (2.3%)	1 (2.4%)	4 (1.2%)	5 (1.6%)	5 (3.8%)
Other	3256 (81.8%)	5319 (60.3%)	1554 (46.1%)	61 (32.1%)	3458 (43.4%)	1149 (41.3%)	499 (37.7%)	2197 (73.8%)	721 (52.9%)	625 (46.5%)	25 (61.0%)	155 (45.1%)	170 (54.8%)	41 (30.8%)

Values are expressed as n (%) unless otherwise indicated. Data are presented as mean (SD) or median (Q1–Q3). AE, adverse event; FAERS, FDA Adverse Event Reporting System; NA, no reports (not missing data); NSCLC, non‑small cell lung cancer; Q1 and Q3, the first and third quartiles, respectively (Q1–Q3 = interquartile range); SD, standard deviation.

#### Descriptive analysis

For PTs identified as key signals, we summarized the characteristics of contributing reports. Descriptive analyses included sex, age, weight, reporter type, outcomes, and indications. Continuous variables were summarized as medians with interquartile ranges (IQRs), and categorical variables as counts and percentages. Temporal trends and geographic distributions were also assessed.

#### Missing-data handling

For missing values in key baseline variables such as sex, age, and weight, we performed multiple imputation under a missing-at-random (MAR) assumption. Imputation was conducted using the *mice* package in R 4.5.1, with m = 5 imputed datasets and a maximum of 30 iterations (maxit = 30). Continuous variables (AGE, WT) were imputed using predictive mean matching (PMM); SEX was imputed using logistic regression; and AGE_COD, WT_COD, and REPORTER_COUNTRY were imputed using multinomial logistic regression (polyreg). REPORTER_COUNTRY and GetDataYear were included as auxiliary variables to better approximate the MAR mechanism.

### Linking key PT signals to SOCs

Using MedDRA v27.0, PT-level signals jointly identified by all four algorithms were mapped to SOCs. For each drug, we extracted all signal PTs (by count “a”), grouped them by SOC, and constructed a drug–SOC–PT atlas to compare the distribution of shared versus drug-specific PTs across agents. Within MedDRA’s multiaxial structure, the SOC *Investigations* typically denotes laboratory or diagnostic abnormalities; concurrent enrichment of Investigations together with a given organ-specific SOC was interpreted as evidence of “marker–organ concordance.”

### Drug similarity network analysis

We constructed a binary drug–PT matrix based on key PT-level signals, with each drug represented by its set of positive PTs. Pairwise similarity between drugs was quantified using the Jaccard coefficient[36]. For any two drugs A and B, J (A, B) was defined as the size of the PT intersection divided by the size of the union. The resulting 14 × 14 similarity matrix was converted to a dissimilarity matrix Qijin Shu and treated as a distance metric.

We generated a similarity heat map to visualize overlap in AE profiles. We also interpreted the similarity matrix as a weighted undirected graph and built a drug similarity network, in which edge weights corresponded to Jaccard similarities. To obtain an interpretable backbone, we retained only edges at or above the 80th percentile of the empirical similarity distribution, thereby focusing on the strongest ≈ 20% of similarities^[37–39]^. Edge width was scaled by similarity. As a sensitivity analysis, we reconstructed networks using the 70th and 90th percentiles.

Finally, we used multidimensional scaling (MDS) with Qijin Shu as input to visualize drug relationships in two dimensions. The first two principal coordinates were extracted to place each drug in a two-dimensional plane. Euclidean distances between points approximate differences in AE profiles, facilitating assessment of within-target clustering and between-target separation. The full methodological details are provided in the Supplemental Digital Content Methodes, available at: http://links.lww.com/JS9/G620.

### Software

All analyses were conducted in R version 4.5.1, including FAERS preprocessing, descriptive analyses, disproportionality signal detection, and similarity/network construction. Key packages included data.table, tidyverse, ggplot2, viridis, sf, rnaturalearth, openxlsx, igraph, and ggraph. All statistical tests were two-sided with a significance level of α = 0.05, and 95% confidence intervals were reported.

## Results

### Descriptive analysis

**Figure 1.**
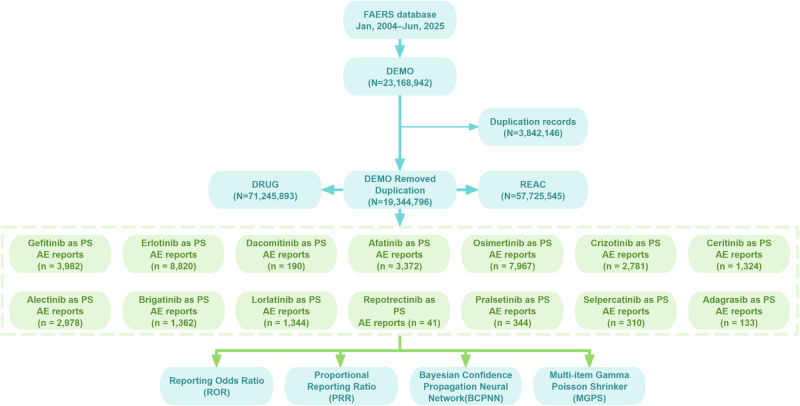
The flowchart of the study.

**Figure 2.**
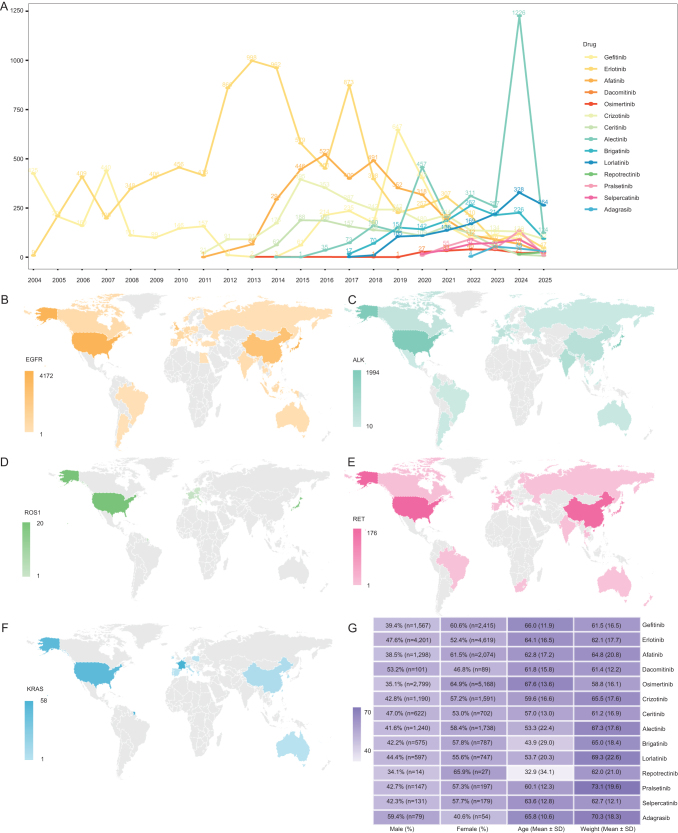
Demographic characteristics of FAERS NSCLC adverse event reports for targeted agents. A: Annual number of adverse event reports for each of the 14 targeted drugs (2004–2025).B: Geographic distribution of reports for EGFR-TKIs.C: Geographic distribution of reports for ALK-TKIs.D: Geographic distribution of reports for the ROS1-TKI (repotrectinib).E: Geographic distribution of reports for RET-TKIs.F: Geographic distribution of reports for the KRAS G12C inhibitor (adagrasib).G: Distribution of sex, age, and weight among adverse event reports, stratified by drug.

The study population consisted of NSCLC cases with reported AEs in FAERS (2004–2025), and the analytic workflow is shown in Figure 1. In total, AE reports involving 14 targeted agents were identified, covering 34 948 individuals. Report volumes varied markedly across drugs: first-generation EGFR-TKIs (gefitinib, erlotinib) peaked around 2013–2019, whereas the third-generation EGFR-TKI osimertinib increased substantially after 2021. ALK-TKIs showed a similar pattern of generational shift, while reports for RET-TKIs and the KRAS G12C inhibitor only began to appear after 2020, with relatively low annual counts (Fig. 2A).

Geographically, AE reports were concentrated in a small number of high-reporting countries/regions, mainly the United States, China, Japan, and the United Kingdom. However, the proportion of missing country/region information was high for some drugs – for example, “NA” (unspecified country/region) accounted for 44.12% and 33.29% of reports for gefitinib and erlotinib, respectively. Accordingly, the geographic distributions primarily reflect the composition of “information-available” cases (Fig. 2B–F). Key baseline variables such as sex, age and weight also had varying degrees of missingness (Supplemental Digital Content Table 2, available at: http://links.lww.com/JS9/G623), and multiple imputation was therefore applied (Table 1). Comparisons before and after imputation showed similar overall distributions, suggesting that imputation did not introduce substantial bias.

**Table 2:** Top 10 preferred term-level disproportionality signals for targeted agents used in non-small cell lung cancer in the United States FDA Adverse Event Reporting System, 2004–2025.

Drug	PT	a	ROR (95%Cl)0	PRR	X[2]	IC(IC025)	EBGM(EBGM05)
Gefitinib	Hepatic function abnormal	165	3.25 (2.76–3.82)	3.22	227.3	1.58 (1.32)	2.99 (2.54)
	Lung disorder	158	4 (3.38–4.73)	3.96	306.81	1.84 (1.57)	3.59 (3.03)
	Liver disorder	115	3.93 (3.23–4.78)	3.9	217.97	1.82 (1.51)	3.54 (2.91)
	Dry skin	70	2.75 (2.15–3.52)	2.74	70.58	1.37 (0.98)	2.58 (2.02)
	Acne	40	3.54 (2.55–4.92)	3.53	64.38	1.7 (1.14)	3.24 (2.33)
	Blood pressure decreased	31	3.18 (2.19–4.62)	3.18	41.46	1.56 (0.93)	2.95 (2.03)
	Depressed level of consciousness	30	3.2 (2.19–4.67)	3.19	40.51	1.57 (0.93)	2.96 (2.03)
	Cystitis hemorrhagic	25	27.51 (15.8–47.9)	27.46	318.73	3.83 (2.52)	14.23 (8.17)
	Dermatitis exfoliative	10	5.72 (2.9–11.32)	5.72	32.24	2.29 (0.89)	4.91 (2.48)
	Laryngeal pain	7	6.2 (2.73–14.09)	6.2	24.91	2.39 (0.63)	5.24 (2.31)
Erlotinib	Rash	1416	3.94 (3.7–4.19)	3.81	2176.05	1.61 (1.52)	3.05 (2.87)
	Dry skin	244	4.83 (4.15–5.62)	4.8	502.87	1.85 (1.62)	3.6 (3.09)
	Dermatitis acneiform	194	4.98 (4.19–5.91)	4.95	415	1.88 (1.62)	3.68 (3.1)
	Acne	116	5.13 (4.11–6.41)	5.12	257.57	1.91 (1.56)	3.76 (3.01)
	Skin exfoliation	92	3.62 (2.86–4.59)	3.61	129.07	1.55 (1.18)	2.94 (2.32)
	Rash pustular	72	7.56 (5.58–10.24)	7.54	236.59	2.26 (1.77)	4.79 (3.53)
	Dry eye	61	5.46 (4–7.45)	5.45	145.42	1.97 (1.47)	3.92 (2.87)
	Conjunctivitis	55	3.83 (2.81–5.22)	3.83	83.94	1.62 (1.12)	3.06 (2.25)
	Eye irritation	52	14.59 (9.57–22.25)	14.57	273.32	2.73 (2.07)	6.64 (4.36)
	Skin fissures	49	4.8 (3.42–6.73)	4.79	100.62	1.85 (1.29)	3.59 (2.56)
Afatinib	Diarrhea	1289	4.78 (4.49–5.08)	4.44	2972.55	1.97 (1.87)	3.91 (3.67)
	Rash	551	2.91 (2.66–3.18)	2.84	595.58	1.4 (1.27)	2.65 (2.42)
	Stomatitis	356	8.05 (7.13–9.08)	7.87	1619.82	2.63 (2.44)	6.19 (5.49)
	Paronychia	254	13.82 (11.84–16.13)	13.6	1904.65	3.18 (2.92)	9.08 (7.78)
	Dehydration	206	2.61 (2.26–3.01)	2.58	181.99	1.28 (1.06)	2.43 (2.1)
	Dry skin	131	5.01 (4.15–6.05)	4.97	346.08	2.1 (1.79)	4.3 (3.56)
	Acne	112	11.47 (9.16–14.37)	11.39	723.46	3.01 (2.61)	8.07 (6.45)
	Dermatitis acneiform	108	5.37 (4.36–6.62)	5.34	312.47	2.19 (1.84)	4.55 (3.7)
	Epistaxis	85	3.11 (2.48–3.91)	3.1	107.59	1.52 (1.16)	2.86 (2.28)
	Nail disorder	58	10.11 (7.44–13.73)	10.07	335.17	2.89 (2.31)	7.41 (5.46)
Dacomitinib	Blood pressure increased	21	27.25 (17.44–42.57)	26.61	487.37	4.65 (2.94)	25.09 (16.06)
	Paronychia	20	12.53 (7.99–19.65)	12.27	201.5	3.58 (2.32)	11.95 (7.62)
	Hemoglobin decreased	13	5.67 (3.27–9.84)	5.6	48.58	2.47 (1.28)	5.54 (3.19)
	Mucosal inflammation	12	5.83 (3.28–10.34)	5.76	46.69	2.51 (1.25)	5.7 (3.21)
	Dry skin	8	4.46 (2.21–8.97)	4.42	21.03	2.13 (0.7)	4.39 (2.18)
	Skin disorder	8	6.52 (3.23–13.14)	6.47	36.47	2.67 (1.02)	6.38 (3.17)
	Nail disorder	8	17.92 (8.81–36.48)	17.77	121.54	4.1 (1.62)	17.09 (8.4)
	Heart rate increased	7	10.66 (5.02–22.65)	10.59	59.33	3.37 (1.21)	10.35 (4.88)
	Weight increased	6	5 (2.23–11.22)	4.97	18.85	2.3 (0.55)	4.93 (2.2)
	Mouth ulceration	6	10.93 (4.85–24.66)	10.87	52.43	3.41 (1.05)	10.62 (4.71)
Osimertinib	Electrocardiogram qt prolonged	115	10.01 (7.95–12.61)	9.96	585.87	2.74 (2.35)	6.66 (5.29)
	Paronychia	106	3.02 (2.45–3.71)	3	120.78	1.44 (1.11)	2.7 (2.2)
	Cardiac disorder	54	4.74 (3.5–6.4)	4.73	124.29	1.97 (1.46)	3.92 (2.9)
	Urticaria	52	3.39 (2.52–4.57)	3.39	73.04	1.58 (1.09)	2.99 (2.22)
	Ejection fraction decreased	51	5.59 (4.07–7.67)	5.58	144.52	2.15 (1.61)	4.45 (3.24)
	Cardiac failure congestive	48	3.18 (2.34–4.33)	3.17	60.34	1.5 (1)	2.83 (2.08)
	Taste disorder	42	4.3 (3.06–6.03)	4.29	84.77	1.86 (1.28)	3.63 (2.59)
	Nail disorder	41	4.46 (3.16–6.3)	4.46	87.19	1.9 (1.32)	3.74 (2.65)
	Cardiomyopathy	40	4.41 (3.11–6.25)	4.4	83.69	1.89 (1.29)	3.71 (2.62)
	Cardiac dysfunction	38	17.09 (10.9–26.81)	17.06	287.39	3.18 (2.32)	9.03 (5.76)
Crizotinib	Edema peripheral	114	3.51 (2.9–4.26)	3.48	185.88	1.71 (1.4)	3.28 (2.7)
	Visual impairment	78	9.13 (7.13–11.69)	9.06	455.31	2.92 (2.44)	7.55 (5.9)
	Peripheral swelling	66	5.48 (4.23–7.1)	5.45	210.94	2.3 (1.84)	4.91 (3.79)
	Edema	53	3.08 (2.33–4.08)	3.07	68.75	1.55 (1.09)	2.92 (2.21)
	Bradycardia	49	8.62 (6.32–11.75)	8.58	269.53	2.85 (2.23)	7.22 (5.3)
	Dysgeusia	47	5.08 (3.75–6.89)	5.06	135.74	2.2 (1.65)	4.6 (3.39)
	Photopsia	33	50.17 (29.99–83.91)	49.99	698.26	4.5 (3.15)	22.59 (13.5)
	Vision blurred	31	4 (2.76–5.79)	3.99	63.13	1.89 (1.24)	3.71 (2.57)
	Dyspepsia	28	3.32 (2.26–4.88)	3.31	41.72	1.65 (0.98)	3.13 (2.13)
	Electrocardiogram qt prolonged	26	3.59 (2.4–5.36)	3.58	44.36	1.75 (1.05)	3.37 (2.25)
Ceritinib	Nausea	203	2.75 (2.38–3.17)	2.68	208.9	1.39 (1.17)	2.62 (2.27)
	Vomiting	160	2.92 (2.48–3.42)	2.86	187.33	1.48 (1.22)	2.78 (2.37)
	Abdominal pain	59	3.29 (2.53–4.28)	3.27	88.61	1.66 (1.22)	3.16 (2.43)
	Blood creatinine increased	49	2.93 (2.2–3.91)	2.91	59.17	1.5 (1.03)	2.83 (2.13)
	Blood alkaline phosphatase increased	31	4 (2.78–5.76)	3.99	65.43	1.93 (1.28)	3.81 (2.65)
	Pericarditis	29	9.33 (6.32–13.78)	9.28	187.8	3.04 (2.17)	8.25 (5.59)
	Hyperglycemia	26	4.75 (3.19–7.07)	4.73	71.38	2.16 (1.41)	4.48 (3)
	Nasopharyngitis	24	4.02 (2.66–6.08)	4.01	51.12	1.94 (1.19)	3.83 (2.54)
	Abdominal discomfort	19	4.04 (2.54–6.42)	4.03	40.78	1.95 (1.08)	3.85 (2.42)
	Hepatic infection	17	111.36 (50.97–243.32)	111.02	686.55	5.38 (2.79)	41.75 (19.11)
Alectinib	Fatigue	198	2.43 (2.1–2.81)	2.39	154.86	1.22 (1)	2.33 (2.01)
	Constipation	168	5.12 (4.37–6.02)	5.03	494.89	2.22 (1.95)	4.66 (3.97)
	Myalgia	107	7.74 (6.31–9.5)	7.64	536.3	2.76 (2.38)	6.75 (5.5)
	Blood bilirubin increased	95	13.58 (10.81–17.05)	13.41	860.25	3.43 (2.96)	10.77 (8.58)
	Edema peripheral	89	3.39 (2.73–4.21)	3.36	138.94	1.68 (1.33)	3.21 (2.59)
	Aspartate aminotransferase increased	79	3.41 (2.71–4.28)	3.38	124.46	1.69 (1.32)	3.23 (2.57)
	Edema	75	5.71 (4.49–7.26)	5.66	259.07	2.37 (1.95)	5.19 (4.08)
	Weight increased	73	8.28 (6.46–10.62)	8.21	397.06	2.85 (2.37)	7.19 (5.6)
	Blood creatine phosphokinase increased	70	7.66 (5.95–9.86)	7.59	348.19	2.75 (2.27)	6.72 (5.22)
	Blood creatinine increased	70	3.25 (2.55–4.15)	3.23	101.49	1.63 (1.23)	3.09 (2.43)
Brigatinib	Blood creatine phosphokinase increased	96	20.67 (16.53–25.84)	20.19	1434.96	4.06 (3.52)	16.7 (13.36)
	Pulmonary toxicity	34	5.3 (3.75–7.5)	5.26	111.17	2.33 (1.67)	5.03 (3.55)
	Amylase increased	28	13.55 (9.1–20.18)	13.46	281.51	3.57 (2.53)	11.85 (7.96)
	Lipase increased	20	12.95 (8.1–20.71)	12.89	192.18	3.51 (2.25)	11.41 (7.14)
	Blood pressure increased	19	5.17 (3.26–8.22)	5.15	60.25	2.3 (1.37)	4.93 (3.1)
	Dry mouth	16	4.85 (2.93–8.02)	4.83	46.18	2.21 (1.21)	4.64 (2.8)
	Somnolence	16	3.59 (2.18–5.92)	3.58	28.67	1.8 (0.89)	3.48 (2.11)
	Muscle spasms	14	4.29 (2.51–7.35)	4.28	33.68	2.05 (1.01)	4.14 (2.42)
	Memory impairment	14	4.91 (2.86–8.41)	4.89	41.18	2.23 (1.14)	4.69 (2.74)
	Photosensitivity reaction	14	12.64 (7.22–22.12)	12.6	131.3	3.48 (1.94)	11.18 (6.39)
Lorlatinib	Blood cholesterol increased	98	209.38 (138.98–315.43)	205.81	4682.95	5.61 (4.65)	49 (32.53)
	Edema peripheral	81	3.9 (3.11–4.89)	3.86	162.17	1.88 (1.51)	3.69 (2.95)
	Weight increased	79	11.56 (9.08–14.71)	11.42	636.48	3.3 (2.79)	9.82 (7.71)
	Hallucination	76	51.07 (37.73–69.12)	50.4	2045.42	4.83 (3.99)	28.45 (21.01)
	Hypercholesterolemia	58	131.83 (83.91–207.12)	130.5	2427.36	5.43 (4.16)	43.16 (27.47)
	Hemoglobin decreased	54	3.65 (2.77–4.81)	3.62	97.24	1.8 (1.33)	3.48 (2.64)
	Blood triglycerides increased	54	190.8 (111.85–325.48)	189.01	2525.16	5.59 (4.17)	48 (28.14)
	Edema	53	4.95 (3.74–6.55)	4.91	153.42	2.21 (1.71)	4.63 (3.49)
	Neuropathy peripheral	46	2.95 (2.19–3.97)	2.94	56.28	1.51 (1.02)	2.85 (2.12)
	Memory impairment	44	12.14 (8.78–16.78)	12.05	374.63	3.36 (2.62)	10.28 (7.44)
Repotrectinib	Dizziness	12	30.73 (16.89–55.94)	27.76	307.89	4.78 (2.33)	27.52 (15.12)
	Paresthesia	4	30.69 (11.27–83.53)	29.7	109.98	4.88 (0.81)	29.42 (10.81)
	Neuropathy peripheral	3	9.09 (2.88–28.64)	8.89	21	3.15 (0.11)	8.86 (2.81)
Pralsetinib	Asthenia	32	3.34 (2.34–4.75)	3.27	50.35	1.7 (1.09)	3.25 (2.28)
	White blood cell count decreased	25	5.8 (3.89–8.65)	5.69	95.41	2.49 (1.67)	5.61 (3.76)
	Hypertension	24	6.68 (4.44–10.05)	6.56	111.27	2.69 (1.81)	6.45 (4.29)
	Myelosuppression	12	4.09 (2.31–7.25)	4.06	27.39	2.01 (0.89)	4.02 (2.27)
	Blood pressure increased	11	10.48 (5.73–19.16)	10.39	90.55	3.34 (1.67)	10.1 (5.52)
	Blood creatine phosphokinase increased	9	5.66 (2.92–10.96)	5.62	33.64	2.47 (1)	5.54 (2.86)
	Mouth ulceration	7	9.83 (4.62–20.9)	9.78	53.57	3.25 (1.16)	9.52 (4.48)
	Chest discomfort	7	6.63 (3.13–14.04)	6.59	32.58	2.7 (0.91)	6.48 (3.06)
	Gastritis	5	6.07 (2.5–14.73)	6.04	20.68	2.57 (0.51)	5.95 (2.45)
	Hepatitis b reactivation	5	45.2 (17.7–115.39)	45	188.9	5.31 (1.15)	39.64 (15.53)
Selpercatinib	Hypersensitivity	23	19.18 (12.57–29.28)	18.66	369.83	4.17 (2.78)	17.96 (11.77)
	Hepatic function abnormal	23	6.87 (4.53–10.43)	6.7	110.46	2.73 (1.82)	6.62 (4.36)
	Hypertension	18	6.95 (4.34–11.13)	6.82	88.35	2.75 (1.69)	6.73 (4.21)
	Alanine aminotransferase increased	15	5.34 (3.19–8.92)	5.26	51.32	2.38 (1.31)	5.21 (3.12)
	Aspartate aminotransferase increased	12	4.46 (2.52–7.91)	4.41	31.43	2.13 (0.98)	4.38 (2.47)
	Liver function test increased	11	23.17 (12.59–42.64)	22.87	219.11	4.45 (2.13)	21.82 (11.86)
	Liver disorder	10	4.99 (2.66–9.33)	4.94	31.13	2.29 (0.97)	4.89 (2.61)
	Hypertransaminasemia	10	19.02 (10.07–35.95)	18.8	161.9	4.18 (1.93)	18.09 (9.57)
	Edema	9	5.66 (2.92–10.96)	5.61	33.73	2.47 (1.01)	5.55 (2.87)
	Ascites	9	12.07 (6.2–23.48)	11.94	88	3.54 (1.56)	11.66 (5.99)
Adagrasib	Vomiting	17	3.63 (2.23–5.89)	3.53	31.01	1.81 (0.93)	3.52 (2.17)
	Agranulocytosis	5	15.99 (6.57–38.95)	15.83	68.15	3.96 (0.98)	15.54 (6.38)
	Abdominal distension	4	10.46 (3.88–28.16)	10.37	33.47	3.36 (0.54)	10.25 (3.81)
	Hepatic cytolysis	4	7.75 (2.88–20.84)	7.69	23.09	2.93 (0.41)	7.63 (2.84)
	Gamma-glutamyltransferase increased	4	6.24 (2.32–16.76)	6.2	17.32	2.62 (0.29)	6.16 (2.29)
	Liver function test increased	4	14.19 (5.26–38.31)	14.08	47.78	3.79 (0.64)	13.85 (5.13)
	Joint swelling	3	11.49 (3.66–36.06)	11.43	28.15	3.5 (0.2)	11.28 (3.59)
	Encephalopathy	3	11.96 (3.81–37.53)	11.89	29.48	3.55 (0.21)	11.72 (3.74)
	Skin discoloration	3	19.19 (6.08–60.54)	19.07	50.18	4.22 (0.31)	18.65 (5.91)
	Status epilepticus	3	76.79 (23.39–252.06)	76.3	203.28	6.12 (0.41)	69.65 (21.22)

Most drugs were more frequently reported in women, particularly EGFR-TKIs, with dacomitinib and adagrasib as the main exceptions (Fig. 2G). Age distributions differed across targets (Supplemental Digital Content Figures 1–3, available at: http://links.lww.com/JS9/G621): EGFR-related reports were mainly in patients aged 65–85 years (with osimertinib skewed toward older ages), ALK-related reports in those aged 18–64 years (with relatively more patients <18 years for alectinib and brigatinib), RET-related reports in an intermediate range, repotrectinib in the youngest patients, and adagrasib in a distribution similar to EGFR-TKIs. For weight, patients treated with EGFR-TKIs tended to have lower body weight, whereas those receiving ALK/RET-TKIs tended to be heavier (Supplemental Digital Content Figures 4–6, available at: http://links.lww.com/JS9/G621). Report sources and serious outcomes differed by drug and class, but given the limitations of spontaneous reporting and confounding by disease severity, line of therapy and other factors, these patterns are considered hypothesis-generating (Supplemental Digital Content Figures 7–8, available at: http://links.lww.com/JS9/G621).

**Figure 3.**
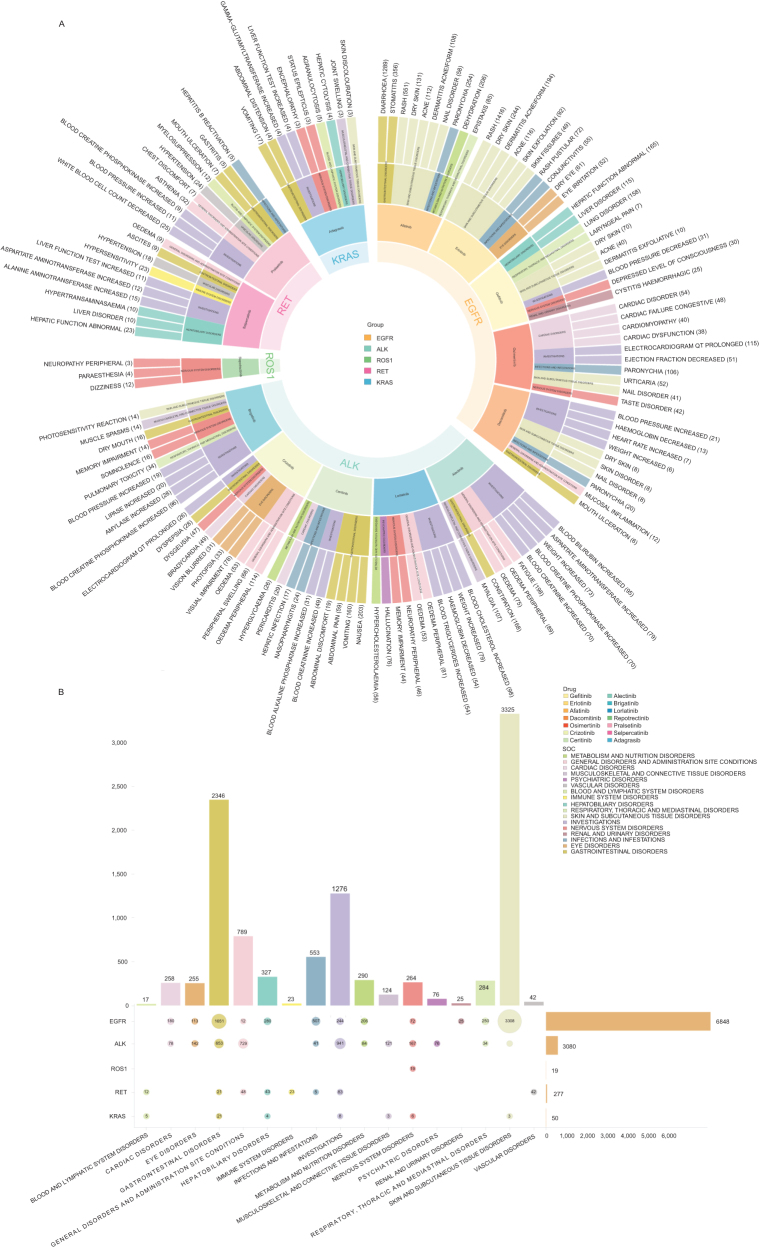
PT- and SOC-level patterns of key adverse event signals for NSCLC targeted agents in FAERS. A: Circular visualization of the top 10 PT-level adverse event signals for each of the 14 targeted drugs. B: Bubble plot showing the distribution of System Organ Class (SOC) categories among the top 10 PT signals, stratified by molecular target class (EGFR, ALK, ROS1, RET, KRAS G12C).

**Figure 4.**
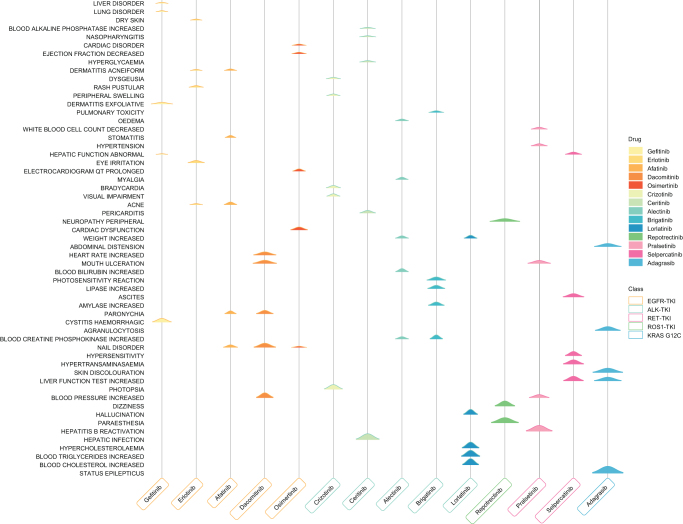
EBGM-based hill plot of the top 5 PT-level adverse event signals for each targeted agent.

### Signal detection and PT-level patterns

At the PT level, we applied four disproportionality methods – PRR, ROR, BCPNN, and MGPS – for signal detection. Among the 14 drugs, the number of key PT signals that met all four criteria ranged from 3 to 113 (Supplemental Digital Content Figure 9, available at: http://links.lww.com/JS9/G621; Supplemental Digital Content Table 3, available at: http://links.lww.com/JS9/G624), and their distributions at the SOC level are summarized in Supplemental Digital Content Table 4, available at: http://links.lww.com/JS9/G625. Subsequent analyses focused on the top 10 key PTs for each drug ranked by report counts, together with their SOC distribution patterns (Fig. 3A, B; Table 2). In addition, based on the key PT signals jointly identified by all four methods, we further ranked them by EBGM values and visualized the top five PT-level signals for each drug (Fig. 4).

#### EGFR class

For EGFR-TKIs, signals were mainly concentrated in skin and subcutaneous tissue disorders, periungual/nail-related events, and investigation-related PTs (e.g., blood pressure, electrocardiogram, body weight), whereas gastrointestinal, hepatobiliary, and respiratory signals were relatively less prominent. Afatinib showed stronger disproportionality for periungual/skin events; gefitinib exhibited a genitourinary-specific signal (cystitis hemorrhagic); and dacomitinib was associated with blood pressure increased and nail disorders. Compared with first- and second-generation EGFR-TKIs, osimertinib, while still linked to paronychia and nail abnormalities, displayed more prominent cardiac and electrocardiographic signals, including QT-interval prolongation and cardiac dysfunction.

#### ALK class

For ALK-TKIs, signals overall clustered in laboratory test abnormalities, metabolic disturbances, and edema-like manifestations, while showing clear drug-specific differences. Crizotinib was characterized by visual AEs and edema with QT-interval prolongation; ceritinib was dominated by hepatotoxicity and pericardial/central nervous system events; alectinib mainly presented with hemolysis, photosensitivity, and multiple laboratory abnormalities (e.g., elevations in bilirubin and CPK), accompanied by weight gain and edema; brigatinib’s principal signals were elevations in CPK and pancreatic enzymes, together with hypertension and pulmonary toxicity; and lorlatinib exhibited a highly distinctive lipid-metabolism toxicity profile, accompanied by weight gain, edema, and notable neurological and psychiatric AEs.

#### RET class

Both pralsetinib and selpercatinib showed signals for liver-function abnormalities, hypertension/blood pressure increases, and laboratory-test-related PTs, together with general symptoms and infection/immune-related AEs. Pralsetinib additionally presented with leukopenia, myelosuppression, CPK elevation, and gastrointestinal toxicities such as oral ulceration and gastritis, with a particularly prominent signal for hepatitis B reactivation. Selpercatinib’s toxicity profile was more focused on hepatotoxicity and blood pressure abnormalities, with frequent liver function test increased, hypertransaminasemia, hypertension, hypersensitivity, and ascites.

#### ROS1 and KRAS

Repotrectinib generated only a small number of neurosensory signals (dizziness, paraesthesia, and peripheral neuropathy); given the limited sample size, these findings should be interpreted with caution and regarded as exploratory rather than definitive. Adagrasib exhibited a multisystem toxicity profile dominated by gastrointestinal and liver-function abnormalities, accompanied by hematologic toxicity, neurological events (including encephalopathy and status epilepticus), joint swelling, and skin pigmentation changes, indicating partial overlap with EGFR/ALK safety profiles while retaining clear drug-specific patterns.

### Cross-class commonalities and differences

**Figure 5.**
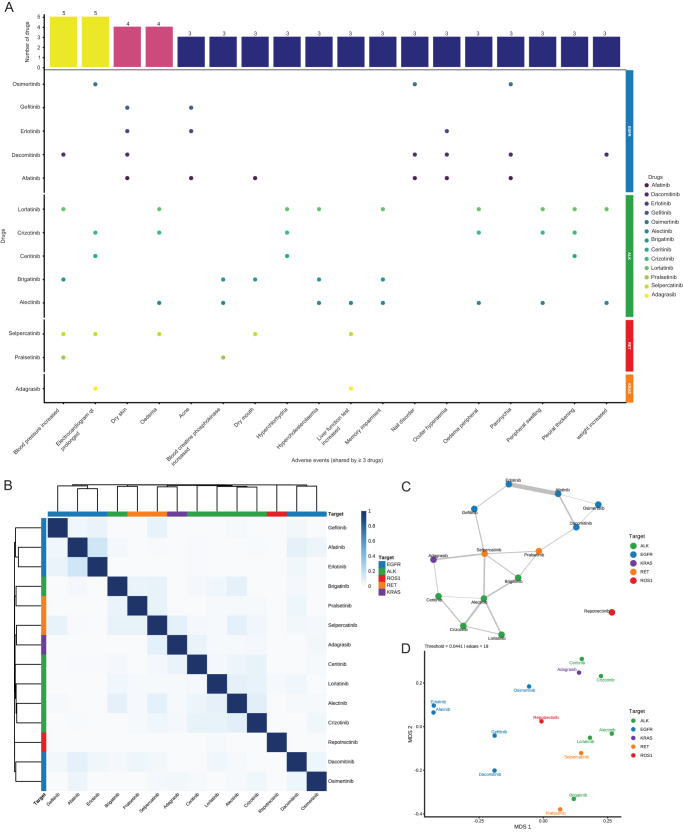
Cross-drug AE overlap and similarity structure. A: AE entries shared across drugs.B: Heatmap – pairwise Jaccard similarity among the 14 drugs; row/column annotations indicate molecular targets.C: Similarity network – edges retained above the top 20% Jaccard quantile; nodes are drugs colored by target, and edge width scales with similarity.D: MDS – 2D layout using distance = 1 − Jaccard similarity; node colors denote targets and labels show drug names, illustrating clustering/separation of toxicity profiles.

In the shared-PT analysis (Fig. 5A), no single PT signal was observed across all 14 drugs, suggesting the absence of a universal “global class effect.” Recurrent cross-category PTs included blood pressure increased and electrocardiogram QT prolonged (each present in five drugs), indicating that these two cardiovascular events may represent key safety concerns shared by multiple TKIs. Dry skin was almost exclusively observed with EGFR-TKIs, forming a characteristic cutaneous toxicity signature, whereas edema patterns were primarily driven by ALK inhibitors. Most of the other shared PTs formed organ-/system-specific clusters within classes (e.g., periungual/skin and mucosal events within the EGFR class; edema-like, metabolic, and neurocognitive events within the ALK class), while cross-class overlap was mainly reflected in muscle-enzyme elevations, liver-function abnormalities, and mucosal dryness.

### Drug similarity clustering and network analysis

The drug similarity matrix based on the Jaccard coefficient showed clear within-class clustering and between-target separation of AE-signal profiles (Fig. 5B). In the similarity network using the 80th percentile of the empirical similarity distribution as the edge threshold, EGFR, ALK, and RET inhibitors each formed distinct modules, whereas adagrasib and repotrectinib consistently lay at the network periphery; sensitivity analyses using the 70th and 90th percentile thresholds confirmed the robustness of this modular structure (Fig. 5C; Supplemental Digital Content Figure 10, available at: http://links.lww.com/JS9/G621). The two-dimensional MDS projection based on Jaccard distance likewise showed tight within-class aggregation and clear separation between different molecular targets (Fig. 5D), supporting the notion that AE-signal profiles can, to some extent, reflect both shared and distinct safety characteristics among targeted agents.

## Discussion

This study used FAERS data from 2004–2025 and four disproportionality methods (PRR, ROR, BCPNN, and MGPS) to describe real-world safety profiles of 14 targeted agents across five molecular subtypes of NSCLC. Overall, EGFR-TKIs remained mainly associated with cutaneous, periungual, and mucosal toxicities. ALK-TKIs were characterized by metabolic disturbances, laboratory abnormalities, and edema-like AEs. RET inhibitors primarily involved hepatobiliary toxicity. ROS1 and KRAS G12C inhibitors showed newer and still evolving toxicity patterns. Across drugs, blood pressure elevation, liver enzyme abnormalities, and QT-interval prolongation emerged as safety concerns that recurred in multiple agents. A similarity network and MDS analysis based on the Jaccard coefficient showed clear clustering of drugs with the same molecular target, while repotrectinib and adagrasib were frequently located at the network periphery. These patterns suggest that both target-related class effects and drug-specific differences contribute to the overall AE spectrum.

EGFR-TKIs are small-molecule oral inhibitors that target the intracellular tyrosine kinase domain of EGFR. Their most frequent AEs involve the skin and gastrointestinal tract, which is consistent with high EGFR expression in the epidermis and gastrointestinal epithelium and with barrier dysfunction induced by EGFR blockade^[40,41]^. The third-generation EGFR-TKI osimertinib has improved survival outcomes in patients with EGFR-mutant NSCLC in several randomized phase III trials^[42,43]^. However, its cardiovascular toxicity requires careful attention. In the AURA3 trial, QT-interval prolongation was reported in 4% of patients treated with osimertinib and in 1% (1/276) of those receiving platinum–pemetrexed[42]. In the FLAURA trial, QTc prolongation occurred in 10% of patients in the osimertinib group versus 5% in the comparator EGFR-TKI group, and grade ≥3 QTc events were reported in approximately 2% and 1% of patients, respectively[43]. A systematic review also identified QT prolongation as one of the most frequent cardiac toxicities associated with osimertinib[44]. In our FAERS-based analysis, six of the top 10 disproportionality signals for osimertinib were cardiovascular. QT-interval prolongation accounted for about 5% of all osimertinib-associated NSCLC AEs that met at least one disproportionality criterion and for more than 15% of those that met all four criteria. These findings support structured baseline cardiovascular risk assessment and regular electrocardiogram (ECG)-based monitoring of QT-related parameters during osimertinib treatment.

In the ALK-targeted subgroup, the overall safety profile tended towards metabolic and laboratory abnormalities and edema-like events. This pattern is in line with findings from previous clinical trials and FAERS-based analyses. Peripheral or generalized edema, visual disturbances, and QT-interval prolongation for crizotinib were all among the top 10 PT signals, reproducing the typical pattern of visual symptoms, edema, and heart rate or ECG changes described in PROFILE 1007/1014 and in the product label^[45,46]^. Recent pharmacovigilance work has also emphasized visual discomfort, transaminase elevations, and ECG abnormalities as important signals for crizotinib[47]. Among third-generation ALK-TKIs, lorlatinib showed a distinct toxicity profile. In this study, its top 10 PTs were dominated by hypercholesterolemia, hypertriglyceridemia, weight gain, and edema, together with peripheral neuropathy, memory impairment, and hallucinations. This profile is consistent with reports from the CROWN trial and real-world cohorts, which describe hyperlipidemia, weight gain, and neurocognitive or psychiatric events as key toxicities^[9,48]^. Second-generation ALK-TKIs (ceritinib, alectinib, brigatinib) showed a different pattern: ceritinib was mainly associated with gastrointestinal toxicity and hepatic dysfunction, alectinib with elevations in bilirubin and creatine kinase, and brigatinib with increases in creatine kinase and pancreatic enzymes, along with pulmonary toxicity and hypertension. These findings broadly agree with safety data from ASCEND-4, ALEX/J-ALEX, and ALTA-1L^[49–51]^. In addition, our analysis suggested several AEs that have been less emphasized in trials and labels, such as ceritinib-associated pericarditis and hepatic infection and brigatinib-associated pulmonary toxicity and memory impairment. These signals illustrate how spontaneous-reporting data can complement randomized trials, especially for low-frequency, delayed, or off-label serious events, and support the need for close monitoring and timely management in clinical practice.

For RET, ROS1, and KRAS G12C-targeted agents, the detected signals were broadly consistent with clinical trial data and previous pharmacovigilance studies, but also indicated several potential emerging risks. For the RET inhibitors selpercatinib and pralsetinib, the top 10 PTs mainly included liver function abnormalities, blood pressure elevation, fatigue, and creatine kinase increases, as well as a signal for hepatitis B reactivation. This pattern is similar to the grade 3–4 hypertension and transaminase elevations reported in LIBRETTO-001 and ARROW^[7,51]^. Recent FAERS-based analyses have also highlighted fatigue, hypertension, cytopenias, and increased liver enzymes as common RET inhibitor-related events^[52,53]^. However, most of those studies did not restrict analyses to NSCLC. In our NSCLC-focused cohort, the pralsetinib-associated hepatitis B reactivation signal was again observed. This finding supports the need for thorough baseline hepatitis B virus (HBV) screening and close follow-up of patients with prior HBV infection when RET-TKIs are used. For the ROS1 inhibitor repotrectinib, almost all top signals were dizziness, paraesthesia, and peripheral neuropathy, consistent with the predominantly mild dizziness, dysgeusia, and paraesthesia reported in TRIDENT-1[54]. The KRAS G12C inhibitor adagrasib showed a multisystem toxicity profile. The top PTs were vomiting, abdominal distension, hepatocellular injury, and γ-glutamyl transferase or other liver function test abnormalities, together with agranulocytosis, encephalopathy, and status epilepticus as rare but clinically important central nervous system events. This pattern is broadly in line with the gastrointestinal symptoms and transaminase elevations described in KRYSTAL-1[55], and with FAERS-based reports that have flagged status epilepticus and other brain disorders as potential adagrasib-related signals[56]. These findings suggest that, beyond gastrointestinal and hepatic monitoring, early recognition of altered mental status or seizure-like symptoms is important, and that prospective studies are needed to further clarify the causal nature of these rare events.

Similarity and network analyses indicated that AE profiles were mainly driven by target-related class effects, with relatively limited overlap between different molecular targets. By contrast, blood pressure elevation and QT-interval prolongation emerged as the most prominent cross-target shared toxicities, linking the EGFR, ALK, RET, and KRAS groups. At the mechanistic level, current evidence suggests that hypertension associated with several targeted agents may arise through activation of the thrombospondin-1/CD47 axis and inhibition of the NO–cGMP signaling pathway, leading to impaired vasodilation, increased peripheral vascular resistance, and subsequent blood pressure elevation^[57–59]^. QT-interval prolongation is more likely related to shared effects on cardiac ion channels, including I_Kr via hERG/KCNH2, together with disturbances in myocardial energy metabolism, which jointly increase the risk of ventricular arrhythmias^[60,61]^. In contrast, repotrectinib was dominated by central and peripheral nervous system signals, and adagrasib by gastrointestinal and hepatic toxicity, with limited overlap with the EGFR, ALK, and RET modules. These patterns more strongly reflect drug-specific safety characteristics of these newer agents.

These findings have practical implications for both clinical care and regulation. In clinical practice, follow-up strategies can be organized according to molecular targets and affected organ systems. For example, for EGFR-TKIs, monitoring should prioritize cutaneous and gastrointestinal toxicities and QT-interval changes; for ALK-TKIs, lipids, muscle enzymes, and blood pressure should be followed closely; for RET-TKIs, HBV screening and regular liver function and blood pressure monitoring are recommended; and for repotrectinib and adagrasib, more detailed neurological history taking and examination may be warranted. When selecting first-line and subsequent regimens, these toxicity profiles should be explicitly weighed. Regimens with higher QT liability should be avoided in patients with high cardiovascular risk, and lorlatinib should be used with caution in patients with marked metabolic risk. From a regulatory perspective, signals such as RET-TKI-associated hepatitis B reactivation and adagrasib-associated seizure-like events should be prioritized for assessment in prospective cohorts and registries and may inform updates to label warnings and formal risk-management plans.

This study has several strengths. First, by requiring concordance across four disproportionality methods, we generated a robust AE signal map for NSCLC that covers five major targeted therapy classes. Second, by using the Jaccard index to construct a similarity network and MDS configuration, we compared class effects and drug-specific patterns from multiple angles. However, several limitations should also be considered. First, although MGPS was applied to improve stability in sparse data and four-method concordance was used to reduce false-positive signals, FAERS still has limited sensitivity for extremely rare events. Second, the analysis was restricted to reports in which the study drug was coded as the “primary suspect,” and key covariates were standardized, but residual confounding from comorbidities and concomitant therapies is likely to persist. Third, even with FDA-recommended de-duplication rules and MedDRA coding checks, some duplicate or inconsistently coded records may remain, and the absence of reliable denominator data prevents estimation of true incidence rates. Fourth, although we included all available FAERS reports from 2004 to 2025, most reports originated from a few high-reporting countries, while data from Africa, Latin America, and other regions were sparse. This imbalance limits the generalizability of the findings to under-reported regions. Overall, the present results should be interpreted as pharmacovigilance signals rather than precise incidence estimates or definitive causal evidence, and they require further confirmation in prospective studies and mechanistic investigations.

## Conclusion

Using FAERS AE reports from 2004 to 2025, this study focused on NSCLC and compared the post-marketing safety profiles of EGFR, ALK, ROS1, RET, and KRAS G12C inhibitors. Building on existing evidence, we constructed and systematically characterized both class effects and drug-specific AE signals across these targeted agents, providing safety data that complement findings from clinical trials. These results support drug-specific safety monitoring in clinical practice, particularly in high-risk populations, and further underscore the importance of pharmacovigilance in guiding treatment decisions.

## Data Availability

The data that support the findings of this study are available from the corresponding author upon reasonable request.
